# Hepatitis E in High-Income Countries: What Do We Know? And What Are the Knowledge Gaps?

**DOI:** 10.3390/v10060285

**Published:** 2018-05-25

**Authors:** Lisandru Capai, Rémi Charrel, Alessandra Falchi

**Affiliations:** 1 EA7310 BIOSCOPE, Laboratoire de Virologie, Université de Corse-Inserm, 20250 Corte, France; 2 Unité des Virus Emergents (UVE), Aix-Marseille Université, IRD 190, INSERM 1207, IHU Méditerranée Infection, 13005 Marseille, France; remi.charrel@univ-amu.fr

**Keywords:** Hepatitis E virus, clinical, transmission, zoonosis, virology

## Abstract

Hepatitis E virus (HEV) is a positive-strand RNA virus transmitted by the fecal–oral route. HEV genotypes 1 and 2 infect only humans and cause mainly waterborne outbreaks. HEV genotypes 3 and 4 are widely represented in the animal kingdom, and are mainly transmitted as a zoonosis. For the past 20 years, HEV infection has been considered an imported disease in developed countries, but now there is evidence that HEV is an underrecognized pathogen in high-income countries, and that the incidence of confirmed cases has been steadily increasing over the last decade. In this review, we describe current knowledge about the molecular biology of HEV, its clinical features, its main routes of transmission, and possible therapeutic strategies in developed countries.

## 1. Introduction

Hepatitis E virus (HEV) is one of the leading causes of acute viral hepatitis worldwide, and is responsible for 20 million infections and 70,000 deaths every year [1].

Currently, all of the HEV strains that have been isolated from humans belong to the species *Orthohepevirus* A, with four genotypes implicated in the majority of cases: genotypes 1 and 2 (HEV-1 and HEV-2) infect humans exclusively, whereas genotypes 3 and 4 (HEV-3 and HEV-4) also infect animals and are zoonotic. Usually, HEV-1 and 2 are associated with epidemic outbreaks of Hepatitis E, while HEV-3 and 4 are associated with sporadic cases. A large number of animals have been found to carry HEV, but pigs are the main reservoir [2]. HEV-3 and HEV-4 are emerging pathogens in high-income countries, most likely because of the consumption of contaminated food [3,4]. However, the possible routes of contamination in industrialized countries are not well elucidated, and unsuspected ones may play significant roles in the epidemiology of the disease [5].

In humans, the majority of HEV-3 cases of infection are asymptomatic, but clinically significant forms exist. A small proportion of infections progress to chronic disease, particularly among those with an immunocompromised status and those who have preexisting liver disease [6]. In high-income countries, risk factors for symptomatic or complicated infection include male gender, age > 45, and preexisting liver disease [7].

Over the past 20 years, HEV has been considered an imported disease in developed countries, but there is evidence that autochthonous HEV infection is underrecognized, despite a steadily increasing incidence [8].

In this review, we mainly describe the current knowledge about molecular biology, clinical aspects, and routes of transmission in developed countries in order to highlight gaps and future directions for research studies.

## 2. Discovery

In 1978, the first described HEV epidemic took place in India, in the Kashmir region [9]. It resulted in an estimated 52,000 cases of icteric hepatitis with 1700 deaths. Symptomatic cases were predominant in young adults, with an increased incidence and severity in pregnant women. Initially, this outbreak was classified as non-A and non-B Hepatitis Epidemics. A few years later, another similar outbreak occurred in a soviet military camp in Afghanistan [10]. Dr. Balayan orally self-administrated the stooled extract of an ill patient, and after a few days endured the same symptoms that had been observed in other patients [10]. Electron microscopic analysis of the stools revealed viral particles that agglutinated with serum from convalescent patients with non-A and non-B hepatitis.

In 1990, partial sequences were characterized [11], which was followed by sequencing the complete genome [12,13]. The sequenced strains were isolated from Asia and Mexico, where the virus was spreading epidemically. The hepatitis was named Hepatitis E, with the E referring to enterically transmitted non-A non-B hepatitis [11,14].

Moreover, a retrospective analysis of various reports describing jaundice epidemics with a higher percentage of infection in pregnant women was used to identify Hepatitis E-like epidemics in the last decade of the 18th century [15].

## 3. HEV, a Naked Virus?

HEV is a small, single-stranded, positive-sense and RNA virus (~7.2 kb) with an icosahedral capsid [12]. For many years, this virus has been described as a non-enveloped virus [16,17,18]; however, recently, another form has been identified. HEV can be “masked” by the membrane of the host cells, and be resistant to antibodies when this form is in blood [19,20]. When HEV particles were released by the cellular exosomal pathway, they appeared to be similar to a “quasi-enveloped” virus. The resistance of the “quasi-enveloped” form is caused by the absence of viral antigens on the surface. These different forms were also found for the hepatitis A virus (HAV) [21].

## 4. Taxonomy

The current taxonomy classifies HEV in the family *Hepeviridae* [3]. Since 2015, this family has been split into two genera: *Orthohepevirus*, which contains four species designated as *Orthohepevirus* A to D, and *Piscihepevirus* (Piscihepevirus A) [22,23]. The genus *Orthohepevirus* A includes eight genotypes (HEV-1 to HEV-8). HEV-1 and HEV-2 have been detected in humans only, whereas HEV-3 and HEV-4 have been detected in humans and several animal species (domestic pig, wild boar, *Cervidae*). HEV-7 was detected in humans and *Camelidae* [24]. HEV-5 and HEV-6 have been detected only in wild boars [25,26,27]. The new genotype HEV-8 could infect humans similar to HEV-7, and comes from Bactrian camels [28].

*Orthohepevirus* B consists of avian viruses, and is divided into four proposed subtypes (I–IV), which are mainly detected in domestic chickens. *Orthohepevirus* C includes two genotypes that have been mainly detected in rats (HEV-C1) and carnivores (HEV-C2). *Orthohepevirus* D strains have been detected in different bat species. The genus *Piscihepevirus* includes a single species (A), cutthroat trout virus, which infects trout, although its pathogenicity and full host range are unknown [29].

## 5. Genome

**Figure 1:**
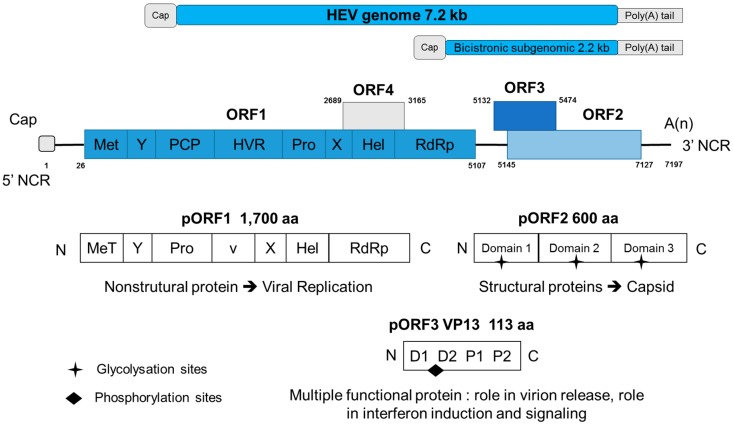
Organization of the Hepatitis E virus (HEV) genome, open reading frames (ORFs), and proteins. Methyltransferase (Met), Y domain (Y), papain-like cysteine protease (PCP), hypervariable region (HVR), proline-rich domain (PRO), X macrodomain (X), RNA helicase (Hel), RNA-dependent RNA polymerase (RdRp), non-coding region (NCR).

The HEV genome is a single-stranded, positive-sense RNA of ~7.2 kb [12], and it is organized as shown in Figure 1. The 5′ cap and 3′ poly(A) tail are non-coding untranslated regions (UTRs). The 5′ cap contains a 7-methyl-guanine. HEV is a positive-sense RNA virus that expresses three open reading frames (ORFs): ORF1, ORF2, and ORF3 [5,30].

ORF1 encodes a set of nonstructural proteins (~1700 amino acids) with eight putative domains: a methyltransferase (Met), a Y domain (Y), a papain-like cysteine protease (PCP), hypervariable region (HVR), proline-rich domain (PRO), an X macrodomain (X), an RNA helicase (Hel), and an RNA-dependent RNA polymerase (RdRp) [1,31]. Enzymes produced from ORF1 are involved in viral replication, transcription, and polyprotein cleavage [11,31,32,33]. It is not yet clear if the polyprotein is cleaved into individual functional domains or it functions as a single protein [34,35].

ORF2 encodes a structural capsid protein. The ORF-translated protein 2 (pORF2) has three glycosylation sites at amino acids (137, 310, and 562). This protein also has an amino-terminal signal peptide that leads to its translocation into the endoplasmic reticulum [36]. ORF2 encodes the structural protein of the icosahedral capsid (660 amino acids) [37], the sequence for which is located at the 3′ end of the RNA strand. It is involved in virus assembly, encapsidation, binding, and the host immune response to the virus [1,38]. Immunological studies of the capsid region have contributed to the development of an HEV vaccine [39].

ORF3 is located between ORF1 and ORF2, and partially overlaps with ORF2 (nt5145–5475) [1]. ORF3 encodes a small phosphoprotein (VP13) of 113 amino acids, 13 kDa [40]. The full role of the VP13 protein is not yet well determined, but studies have been conducted, and it is a multiple functional protein. VP13 is capable of binding to the mammalian cytoskeleton (microtubules) [41]. The ORF3 protein is suggested to act as an adapter to link intracellular transduction pathways, reduce the host inflammatory response, and protect virus-infected cells [42]. VP13 plays an essential role in infectivity in vivo among animal models infected experimentally [43,44]. However, this protein is not indispensable for HEV replication in cultured cells [44]. VP13 is in interacting with different proteins in cells [45]; it plays a role in interferon induction and signaling [46,47,48], and is also involved in virion release [49,50].

A new ORF was discovered on the ORF1 of genotype HEV-1. For this genotype, an additional ORF exists: the ORF4 [51]. The protein encoded by ORF4 is expressed when a stress of endoplasmic reticulum occurs and can stimulate polymerase activity in interaction with an eukaryotic elongation factor [51]. Translation of ORF4 is driven by an internal ribosome entry site IRES-like sequence situated in nucleotide 2701–2787 of HEV genome [51]. The ORF4 product will form a complex with other proteins to stimulate RdRp activity and increase viral replication.

## 6. Putative Replication Cycle of HEV

**Figure 2:**
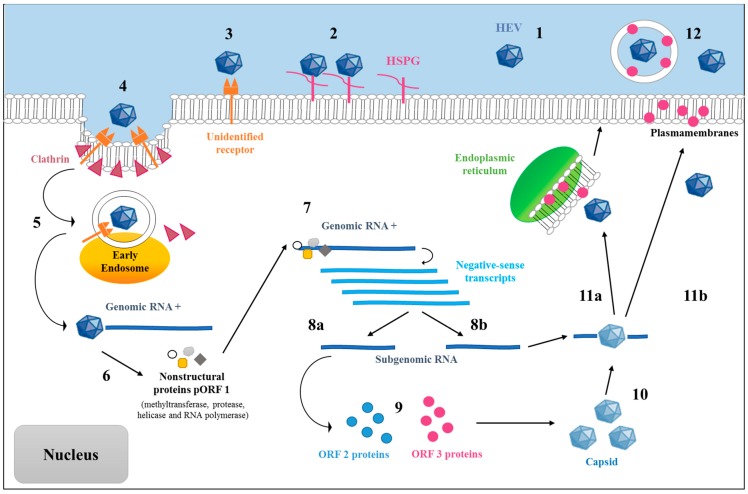
Putative replication cycle of HEV. Step 1: HEV in the extracellular environment. Step 2: Attachment of HEV to the target cell involving heat shock protein. Step 3: Receptor binding. Step 4: Clathrin-dependent endocytosis. Step 5: Encapsidation and liberation of genomic RNA. Step 6: RNA translated into nonstructural ORF-translated protein 1 (pORF1). Step 7: Replication of RNA+ into negative-sense transcripts. Step 8a: Synthesis of subgenomic RNA. Step 8b: Synthesis of full-length positive sense transcripts. Step 9: Translation of subgenomic RNA into ORF2 and ORF3 proteins. Step 10: Capsid formation and assemblage of new virions. Step 11a and 11b: Exit of the virus via the ORF3 proteins fixed on the endoplasmic membranes or the cell wall. Step 12: Mature virions attached to ORF3 protein and lipids (quasi-enveloped form in bloodstream) or free (in bile).

Systems to cultivate HEV in vitro have been developed only recently, and the mechanism of viral replication remains hypothetical. An example of the putative cycle is presented in Figure 2 [52]. Studies including genome analyses and analogies with other known RNA viruses have made it possible to learn more about the replication of HEV. Unsurprisingly, the virus multiplies mainly in hepatocytes (the predominant cells in the liver), but a replication intermediate, viral RNA with negative polarity, has also been detected in the colon, intestine, and lymph nodes of pigs [53], as well as in the cerebrospinal fluid and heart of infected humans [54].

The cellular receptor for HEV is not yet known, but the presence of heparan sulfate proteoglycans appears to be necessary for the binding of the virus to target cells [55]. Additionally, experiments investigating ORF2 peptide binding suggested that the C-terminal region of ORF2 would allow the entry of the virus through binding to heat shock cognate protein 70 (Hsc70) on the cell surface [56].

Once it has bound, HEV enters the cell by endocytosis, which appears to be dynamin-2, clathrin, and membrane cholesterol-dependent [57,58]. The pathway of the virus within the cell remains unknown, but heat shock protein 90 (Hsp90) and tubulin could play a role in the intracellular transport of HEV virions [59]. Subsequently, the RNA is released in the cytoplasm of the cell, but the mechanisms and exact localization of this process have not been explored. Once the viral RNA is free in the cytosol, translation of the ORF1 polyprotein will occur because of its 5′ cap. Following the translation of methyltransferase, protease, helicase, and RNA polymerase activities, the genomic RNA is copied into a strand of negative RNA, which then allows the synthesis of genomic and subgenomic RNAs [60].

Rehman et al. have shown that the 3′ end of the genome binds specifically to the viral replicase on the surface of the endoplasmic reticulum [61]. ORF2 and ORF3 are translated subsequently to produce the structural proteins (capsid protein and phosphoprotein) that will allow the encapsidation of the newly synthesized viral RNA strands. This makes it possible to obtain new viral particles [40,62]; a glucose-regulated protein belonging to the Hsp70 family (Grp78) may play a role in the folding and assembly of the capsid proteins [63]. The proteins encoded by ORF2 and ORF3 interact, which suggests a role for ORF3 in the assembly of viral particles [64]. HEV is not cytolytic [65]. Moreover, studies have shown the requirement of the exosomal pathway for virion release [66,67]. Recent studies suggest that viruses secreted into the bloodstream are associated with the ORF3 protein and lipids, but viruses secreted into the bile are non-enveloped [66,68].

Recently, a new cell culture system has been developed that allows the detection of viral replication and protein expression very early post-transfection [69]. Time-course experiments showed that the ORF2 protein was produced early, and that large amounts were secreted into the supernatant of transfected cells. Interestingly, ORF2 and ORF3 proteins in cell lysate and cell supernatant migrated differently, indicating that these proteins likely undergo post-translational modifications during secretion.

## 7. Clinical

**Figure 3:**
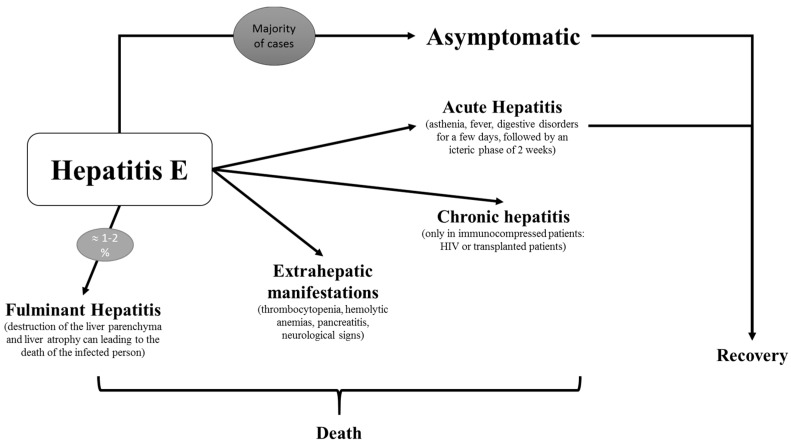
Different clinical forms of Hepatitis E.

The course and clinical presentation of HEV infection is highly variable (Figure 3); the mechanisms leading to the different clinical outcomes are only partially understood [70,71,72,73].

### 7.1. Acute Hepatitis

In humans, the acute form of the disease can be caused by strains belonging to four genotypes: HEV-1, HEV2, HEV3, and HEV-4. Symptoms are resembling those of hepatitis A. Clinical manifestations are similar in developing and industrialized countries [70]. The incubation period ranges from 15 days to nine weeks (mean 40 days). The prodromal phase is quite variable, and can manifest as asthenia, fever, and digestive disorders for several days, followed by an icteric phase of two weeks; accordingly, it is not surprising that most cases remain undetected at the acute stage. Hepatitis is caused by an immune reaction directed towards the infected hepatocytes. Acute cytolytic hepatitis is the most common symptom. In most cases, the outcome is favorable, and biological parameters normalize within three months [74]. Cholestatic forms occur in 20% of cases [75].

Routine laboratory testing usually detects an increase in alanine and aspartate aminotransferase (ALT, AST) levels, accompanied by an increase of alkaline phosphatase (AP), gamma-glutamyl-transferase (γGT), and bilirubin levels. The ALT level increases usually between 1000–3000 IU/L, but extreme values can be seen. ALT elevation is commonly higher than AST elevation. Cases where ALT is normal despite HEV RNA is detected during the acute stage have been described [76].

In industrialized countries, symptomatic HEV infections mostly affect men older than 55 years [77]. The mortality rate is 1–4%, which is higher than the mortality associated with acute hepatitis A (0.1–2.5%) [5]. However, these rates are likely overestimated, because they were calculated from symptomatic cases seen in hospitals. In the general population, the mortality rate ranges between 0.06–0.7% [78]. Cases leading to death correspond to the acute forms, which can become fulminant.

### 7.2. Fulminant Hepatitis

Occasionally (1–2% of cases), acute hepatitis can develop into fulminant hepatitis [79]. Fulminant hepatitis is frequent among people with underlying liver diseases in high-income countries after HEV infection [80]. Cases have been reported in several industrialized countries: the first cases were reported in Italy [81], Spain [82,83,84], France [85,86], and Japan [87,88]. Despite clinical specificities, there would not be a correlation between the severity of the disease and the genotype [89]. However, a case study in France showed that infection with genotype 4 could be more severe [90]. The limits of these studies are the small number of cases, so other studies are needed to clarify this point.

### 7.3. Chronicity

Chronicity is defined as a persistent viremia at least three to six months after the diagnosis [91]. AST and ALT are less elevated in patients who progress to chronic HEV infection; the mean ALT is 300 IU/L in chronic disease, and 1000 IU/L in acute disease [92]. There is no correlation between the viral load and the risk of progression to fibrosis [92].

Although the routes of infection (zoonotic transmission, consumption of infected products) do not differ between the general population and immunocompromised individuals, and the latter can also get infected via blood products or organ donation: transfusion and transplantation-associated cases have been described [93].

The majority of HEV chronic infections is observed with HEV-3, probably because it is the most commonly circulating genotype in industrialized countries [18,94,95,96,97,98,99].

However, chronic infections caused by strains belonging to genotype HEV-4 have been recently described [1,100]. Rapid evolution towards cirrhosis and graft rejection were observed. Cases have been reported in several industrialized countries [101,102,103,104,105].

### 7.4. Extrahepatic Manifestations

Many types of extrahepatic manifestations were reported in both acute and chronic infections [106]; among others, thrombocytopenia, kidney injury, hemolytic anemia, and pancreatitis were described [107,108,109]. Neurological signs are seen in 5% of cases [110,111]: Guillain–Barré syndrome (GBS), neuralgic amyotrophy, and encephalitis/meningoencephalitis/myositis were associated with acute forms [109]. Finally, HEV superinfection can aggravate previous liver diseases caused by alcohol, hepatitis C, or hepatitis B viruses [112,113]. Superinfection must be evoked in the presence of a brutal marked elevation of AST and ALT, or in the case of hepatic encephalopathy or renal impairment.

### 7.5. HEV Infection during Pregnancy

The majority of clinical studies and cases in pregnant women come from developing countries (central Africa and South East Asia, mostly) with genotype 1 and 2. In these highly endemic areas, mortality and vertical transmission rate is high, and severe forms occurred [5,114,115,116,117,118,119].

However, there are few cases reported during pregnancy in industrialized Western countries [120,121,122,123,124]. The first case reported of a pregnant woman infected by HEV with Gt3 in Europe was in a 41-year-old woman living in France [121]. This woman and her baby had no complications. A prospective study in France showed that, out of the 315 pregnant women participating, HEV prevalence was 7.74% [125]. HEV-3 and HEV-4 do not appear to cause fatal infections with fulminant hepatitis in pregnant women [120].

## 8. Different Routes of Transmission

**Figure 4:**
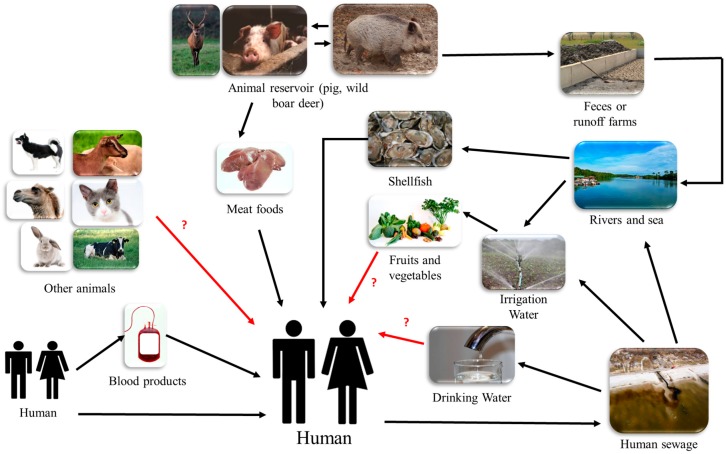
Diagram summarizing the different routes of transmission of HEV in high-income countries. Black arrows mean “confirmed transmission routes” and red arrows with “?” mean “suspected and not confirmed transmission routes”.

As a consequence of the high seroprevalence of HEV in industrialized countries, many questions arise about whether the consumption of contaminated pork is the unique source of infection. There is a growing interest for addressing alternative routes of infection linked with water, other animals, vertical transmission, and transfusion. Figure 4 summarizes all of the transmission pathways described or suspected in the literature.

### 8.1. Zoonotic Transmission

In industrialized countries, the infection of humans with HEV is believed to be zoonotic through the consumption of contaminated foods or by direct contact with infected animals.

In France, a correlation between HEV infection and consumption of the pork liver sausage, ficatellu, has been reported [126,127,128]. Ficatellu is a liver product that contains at least 30% pork liver, and does not undergo a heating step during production. One survey that analyzed food products in France showed that 30% of ficatelli samples were positive for the presence of HEV RNA [129]. In the Netherlands, the rate of HEV RNA was lower than in France, with a detection rate of 6.4% in commercial porcine livers [130]. The HEV RNA sequences were reported to have 93% homology with those detected in human HEV infection in the Netherlands, and 97% homology with previously isolated sequences from swine HEV infections. In Germany, raw and liver sausages were screened for HEV RNA, and 14 of 70 raw sausages (20%) and 11 of 50 liver sausages (22%) were positive. The detected HEV strains belonged to genotype 3, and showed high genetic variability [131].

A survey in the United States (USA) [132] demonstrated that the consumption of undercooked meat was associated with a higher rate of seropositivity for anti-HEV antibodies in college students. Moreover, three of 128 packages of food analyzed in Virginia were positive for HEV RNA (2.3%). In Australia, 55 cases of HEV infection were confirmed [133]. Of these, 24 people had not traveled during the incubation time, and 17 of these had eaten in the same restaurant. The HEV RNA detected was of genotype HEV-3, and 99% homology was found between the restaurant strain and that which was found in confirmed cases.

In Japan, an investigation was conducted in a patient who was infected by HEV after eating wild boar meat [134]. HEV-3 RNA was detected in his serum, in his daughter’s serum, and in the wild boar meat. Venison consumption was repeatedly reported among HEV infected patients; confirmation of the source of infection was provided [135]: the patients ate venison a few weeks before the onset of symptoms, and the HEV RNA that was found in patients had the same sequence as that found in the leftover portions of venison. The virus has also been found in more surprising products such as herbs and spices: among the 230 samples tested, two were positive for HEV RNA [136].

In Switzerland, the prevalence of HEV RNA in 160 pig livers from different farms was 1.3% [137]. Another study had screened ready-to-eat meat products for Hepatitis E virus. Positive rates of 18.9% for liver sausages and 5.7% for raw meat sausages were estimated [138].

However, given the very high prevalence in regions where it is not common to eat uncooked or undercooked products containing pig liver or pig meat, it is legitimate to pursue investigations aiming at documenting other routes of infection.

### 8.2. Waterborne Transmission

The waterborne transmission of HEV (HEV-1 and HEV-2) occurs mainly in developing countries where wastewater treatment is inadequate. Since HEV exist in its non-enveloped form, it is resistant in the environment, and therefore, water-borne transmission is also possible in developed countries when untreated water is ingested.

#### 8.2.1. Irrigation Water

The quality of irrigation water used in several countries in the production chains of fruits and vegetables was estimated [139]. Samples of irrigation water were collected in Finland, Poland, Serbia, and the Czech Republic (*n* = 108), and from three leafy green vegetable farms in Poland, Serbia, and Greece (*n* = 61). The two major origins of contamination of samples used to irrigate berry fruits were fecal contamination from human (8.3%, 9/108) and porcine (4.5%, 4/89). The samples were analyzed for the presence of several viruses, and HEV was identified in one country (5%, 1/20). HEV has also been found in frozen raspberries (2.6%) [140] and on field-grown strawberries [141]. The genotype identified was porcine genotype HEV-3. Irrigation water was the main suspect as the origin of contamination. A metagenomic assessment of the viral contamination of fresh parsley plants in Spain demonstrated the presence of HEV-3f [142]. Among samples from fresh lettuce points of sale, HEV prevalence was 3.2% (4/125) [143].

In Turkey, a study [144] compared farms using treated water (control group) with those using untreated water (test group) for irrigation. The seroprevalence of HEV IgG that was observed among people working on the test group farms (and their families) was higher (34.8%) than that in people working on the control group farms (4.4%).

These reports indicate a possible route of contamination for vegetables and fruits irrigated with contaminated water or food contaminated during the food production chain. Indeed, some vegetables and fruits are consumed raw, which means that even if they are well washed, the virus could still be ingested by consumers.

#### 8.2.2. Contaminated Environmental Water

In Italy, a study analyzed different types of samples (bivalves, water, and raw sewage) for hepatitis A virus and HEV [145]. HEV RNA was found in one sample of raw sewage and in one river sample; both isolates were HEV-3, and their sequences were similar to the human and swine sequences previously defined.

In Slovenia, using seroprevalence data obtained from a pig farm, 60 surface water samples were tested for HEV RNA, of which two were positive (3.3%); one positive specimen had been collected in the vicinity of a pig farm [146]. In Japan, HEV-3 RNA was detected in two of 32 bivalves collected in rivers [147]. In the Netherlands, 17% of surface water samples analyzed were positive for HEV RNA, which probably came from sewage [148]. After a person was infected, viral RNA was detected in the patient’s serum and in the surface water collected in the vicinity of the patient’s house [149].

#### 8.2.3. Filter Feeders: Evidence of Water Contamination and Possible Route of Foodborne Transmission

Water can be contaminated by human wastewater or animal reservoirs, and indirectly infect animals living in water. The rate of HEV RNA detection in filter feeders such as mollusks ranges from 15% in Spain [150] to 85% in the United Kingdom [151]. HEV-3 was detected in two of 32 samples of bivalves mollusks collected from Japanese rivers [147]. In Spain, HEV detected in mollusks were genetically most closely related with viruses circulating in humans and swine. The infection of humans through the consumption of seafood has not been demonstrated, but several studies have pointed to mollusks and shellfishes consumption as probable routes of infection [152].

Although the presence of HEV is undisputable in the environment, low viral loads engage for performing infectivity studies to assess or infirm the role of water as a vector of epidemiological significance.

### 8.3. Risk of Transfusion-Related Transmission

The rate of HEV RNA blood products ranged from 1/726 in the Netherlands [153] to 1/74,131 in Australia [154]. Table 1 shows the rate of blood donations positive for HEV in different industrialized countries. The mean proportion positive was 0.0002 (range, 0.0001–0.0003), i.e., one positive for 5000 donations (1/5000).

A case-control study reported the transmission of HEV by blood in transfused patients [171]. Markers of acute HEV infection were detected significantly more frequently in patients having received blood products (13/145) than in controls (2/250). Three susceptible transfused patients (seronegative for anti-HEV IgG) were infected with HEV, but no controls. Moreover, cases of associated HEV infections have been documented [105,172,173,174,175,176].

A recent study showed that the risk of HEV transmission to blood product recipients correlated with rates of positive blood donations, the viral load, and the volume of plasma in the final transfused blood component [177].

### 8.4. Sexual Transmission

Overall, very little data is available on Hepatitis E, unlike what is described for Hepatitis A. Men having sex with men (MSM) and HIV-infected MSM have a higher prevalence of HEV IgG than the general population in the same region [178,179,180,181,182,183]. So far, there is no direct evidence of transmission by sexual contact. However, in light of the recent findings showing that sperm is an excellent sanctuary for Ebola virus, Zika virus, and dengue virus, with documented cases resulting sexual transmission, such studies should also be performed for HEV.

### 8.5. Other Routes of Transmission

Few studies have been made concerning vertical transmission between mothers and their infants. High transmission rates were described, but these have so far been documented only in India; these were caused by HEV-1: 33.3% (6/18) [116], 46.09% (59/128) [184], and 78.9% (15/18) [185]. Another possible route of HEV transmission between mother and child was demonstrated by Ramos-Juarez et al. [186], who isolated HEV from the breast milk of a 34-year-old woman with HEV infection. In industrialized countries, the rare cases of infection reported in pregnant women (HEV-3) did not lead to infection in the newborn [120,121,122,123]. This genotype specificity deserves to be explored.

Nosocomial transmission of HEV seems very infrequent in industrialized countries. Indeed, very few cases were reported by this route. However, in France, a patient-to-patient transmission of Hepatitis E was described in a hematology ward [187]. The reported case became infected after contact with an undiagnosed chronically infected patient who resided in the same unit. HEV is very resistant, so important hygiene measures must be implemented when a case of Hepatitis E occurs in a unit, and especially when an at-risk patient is present.

## 9. Infection Markers and Diagnosis

The diagnosis of Hepatitis E by the study of clinical signs is quite complicated, because the majority of cases are asymptomatic, and the clinical forms are not specific. The detection of infection markers is therefore required to diagnose Hepatitis E or previous exposure to HEV. Different markers of HEV infection exist: anti-HEV IgG and IgM antibodies, viral genome in blood and body fluids, and alanine aminotransferase activity.

The anti-HEV immunoglobulin M appears first in the kinetics of post-infection markers. Peak detection occurs on average at the eighth week, and IgM are detectable up to 32 weeks after infection [188]. The detection of these IgM antibodies is a marker of recent infection, as is the presence of the viral genome in the blood and stool. The anti-HEV immunoglobulin G appears just after the IgM, and can be detected for years after the infection (immune memory) [189]. IgG are markers of an older infection. However, many studies have shown a very great heterogeneity between commercial tests for Hepatitis E virus IgG antibodies detection [190,191,192,193]. The Wantaï HEV-antigen (Ag) ELISA(Plus) assay for diagnosing acute HEV infections performed better than the others, with better sensitivity and specificity [194]. Moreover, it presented no sensitivity difference for diagnosis between immunocompetent and immunocompromised patients.

A viremia is observed a few days before the appearance of clinical manifestations, and disappears one to two weeks after it [195]. The amount of virus found varies by individual, but according to a study of 11 people in 2007, the viral load ranged from 2 × 10^3^ to 1.7 × 10^7^ genome copies per milliliter [196]. The presence of HEV in the stool is longer, as excretion begins one week before the onset of symptoms, and may last up to six weeks afterwards. However, in immunocompromised patients, this secretion can be considerably longer [102]. Stool viral load is in the range of 5.7 × 104 genome copies per milliliter [196]. The detection of the HEV genome is highly sensitive and specific for the diagnosis of Hepatitis E. The most commonly used technique is real-time reverse transcription PCR (qRT-PCR). It is therefore done from blood samples or stool, and the sequences targeted for the detection of HVE genome are ORF2 and ORF3.

Other marker of Hepatitis Exist, alanine-aminotransferase activity (ALAT) appears two to three weeks after infection with a peak in the sixth week, at the same time as the beginning of the icteric phase [197]. This enzyme activity is not specific for Hepatitis E, but shows symptoms in the liver. ALAT are moderately elevated in immunocompromised patients.

## 10. Treatment, Prevention, and Vaccine

Ribavirin and pegylated alpha interferon show some efficacy against HEV [65]. There is no recommendations for treating HEV infected patients with antiviral regimens. However, a combination of ribavirin and pegylated alpha interferon improved the condition of treated patients. The rapid clearance of HEV viremia permitted avoiding liver transplantation, although the absence of a control group prevents definitive conclusions. Interferon is not used in transplanted patients because of the risk of acute graft rejection.

A multicenter study has shown that ribavirin may be effective in the treatment of chronic HEV infection; indeed, a three-month course seemed to be a good duration of therapy for most patients. A sustained virologic response (SVR) occurred in 46 of the 59 patients (78%) [198]. When patients are under immunosuppressive therapy, reduction of the doses are primarily attempted, since curation is observed in 32% of cases [199]. Durable HEV clearance is observed in 85% of patients after three months of monotherapy, and can be pursued for another three months if viremia remains positive after the first three-month cure.

Liver transplantation is considered when there is fulminant hepatitis and the patient’s condition is life-threatening.

While the main origins of HEV infection are known, several nonspecific means of prevention can be implemented. First, the treatment of wastewater and the quality of water in developing countries remain a major public health problem; untreated water is clearly a source of infection in both developing and industrialized countries. Effective hand washing and correct preparation of “at-risk” foods (heated to >70 °C at the core) are also a means of prevention [128,200,201]. For pregnant women at high risk of fulminant hepatitis, the consumption of “at-risk” foods is strongly discouraged.

Despite the obvious risk of HEV transmission, prevention strategies are currently insufficient and infrequently implemented. Several studies have been undertaken to develop a human vaccine against HEV. Shrestha et al. [202] made a recombinant vaccine and obtained 95.5% efficacy with three doses. Nevertheless, this study did not evolve after phase two. Another study undertaken with more than 50,000 participants [203] showed a vaccine efficacy of 100% after 12 months and 86.8% (95% confidence interval 715–945) after a prolonged follow-up of 4.5 years [204]. People who received three doses (0, one, and six months) maintained their antibodies against HEV for at least 4.5 years. Currently, this vaccine against HEV has obtained approval from the Chinese government [204]; no vaccines are currently available in industrialized countries where HEV transmission is predominantly zoonotic. Therefore, it might be possible to reduce the risk of infection by eliminating the circulation of the virus in the most affected reservoir: pigs. In a modeling study, the effect on the dynamics of virus infection of a fictitious vaccine against late or early zoonotic porcine HEV was assessed. Later vaccination at 10 weeks reduced the proportion of infected pigs at the age of slaughter [205]. Therefore, vaccination of the main reservoir could be the optimal choice. Due to the large numbers of animals to vaccinate and the age at which they are slaughtered, the use of inert vaccines is not applicable. Inactivated vaccines are expensive to produce, and require at least two injections for effective protection. It would be better, in this context, to develop an attenuated live vaccine strain; this is not only cheaper to produce, but a single injection also provides protection similar to that seen after a natural infection [206].

However, the production of live attenuated vaccines is still based on empirical techniques that do not allow fine control or stabilization of the attenuation phenotype. Molecular determinants of attenuation are most often linked to a small number of protein changes. This means that there is a risk of phenotypic reversion, or in contrast, the protein changes can introduce new pathogenic traits that are different from those of the wild-type strain. Moreover, the methods that have been used to produce these vaccines do not make it possible to completely control the composition of the viral subpopulations between different vaccine batches.

## 11. What Do We still Need to Know?

- Even if zoonotic transmission has been identified as an important risk factor in high-come countries, the detection of HEV has been reported in several studies performed in environmental and irrigation water, sewage, and filter feeders. These results arise questions about the role of HEV in other routes of transmission and about its denomination as an environmental virus.
- The epidemiology of HEV in high-income countries is difficult to understand because of the small number of studies, the heterogeneity between them caused by the different assays used, and the type of population included. Gaps related to different suspected animal reservoirs, including food other than pork, need to be explored in order to improve the control and prevention of HEV.
- About the clinical part, the transition to chronicity and the real numbers of symptomatic cases are still poorly understood. Studies could be conducted to deepen knowledge.
- Concerning the replication cycle of HEV, many points are still unknown, especially on the HEV receptor cell and on the exact role of the pORF3 protein. Cell culture progress and discoveries in these areas could lead to advances in treatment and vaccines. A HEV vaccine is currently available in China, but not in other countries worldwide. The development of a vaccine in pigs could reduce the spread of the virus between species and humans.
- The discovery of “quasi-enveloped” HEV particles in blood raises questions about the effects on infectivity, immunity, transmission, and the virus replication cycle.
- Regarding prevention, despite the growing number of cases, Hepatitis E remains unknown to the general public. The population could be sensitized, particularly in the hyperendemic regions. Risky behaviors may thus decrease, especially among people at risk.

## Figures and Tables

**Table 1:** Rate of HEV detection in blood donations in different industrialized countries. USA: United States.

Country	Rate of Positive Blood Products	References
Australia	1/14,799; 1/74,131	[155]
Austria	1/8416	[156]
Denmark	1/2231	[157]
England	1/7040; 1/2848	[158,159]
France	1/2218; 1/744	[160,161]
Germany	1/1240; 1/4525	[162,163]
Ireland	1/4997	[164]
Japan	1/8173	[165]
Netherlands	1/2700; 1/726	[166]
Poland	1/1266	[167]
Scotland	1/14,520	[168]
Spain	1/3333	[169]
Sweden	1/7986	[111,163]
USA	1/9500; 0/51,075; 1/42,674	[170]
